# Affinity-based proteomics reveals novel binding partners for Rab46 in endothelial cells

**DOI:** 10.1038/s41598-021-83560-y

**Published:** 2021-02-18

**Authors:** Lucia Pedicini, Sabina D. Wiktor, Katie J. Simmons, Ashley Money, Lynn McKeown

**Affiliations:** Leeds Institute of Cardiovascular and Metabolic Medicine, Faculty of Medicine and Health, University of Leeds, Leeds, LS2 9JT UK

**Keywords:** Biochemistry, Cell biology

## Abstract

Rab46 is a novel Ca^2+^-sensing Rab GTPase shown to have important functions in endothelial and immune cells. The presence of functional Ca^2+^-binding, coiled-coil and Rab domains suggest that Rab46 will be important for coupling rapid responses to signalling in many cell types. The molecular mechanisms underlying Rab46 function are currently unknown. Here we provide the first resource for studying Rab46 interacting proteins. Using liquid chromatography tandem mass spectrometry (LC–MS/MS) to identify affinity purified proteins that bind to constitutively active GFP-Rab46 or inactive GFP-Rab46 expressed in endothelial cells, we have revealed 922 peptides that interact with either the GTP-bound Rab46 or GDP-bound Rab46. To identify proteins that could be potential Rab46 effectors we performed further comparative analyses between nucleotide-locked Rab46 proteins and identified 29 candidate effector proteins. Importantly, through biochemical and imaging approaches we have validated two potential effector proteins; dynein and the Na^2+^/ K^+^ ATPase subunit alpha 1 (ATP1α1). Hence, our use of affinity purification and LC–MS/MS to identify Rab46 neighbouring proteins provides a valuable resource for detecting Rab46 effector proteins and analysing Rab46 functions.

## Introduction

Rab proteins are the largest member (65 in human) of the Ras superfamily of small guanosine tri-phosphatases (GTPases)^1^. Rabs are master regulators of intracellular vesicle formation, transport and fusion and their importance is evident by the many human diseases caused by mutations that affect these functions^2,3^. Similar to other GTPases, Rabs cycle between a GDP-bound OFF state and a GTP-bound ON state^4^.The GTP-bound state ensures their location at the correct membrane locale where they can interact with effectors such as SNAREs and motor proteins. However, most Rabs lack the inherent ability to efficiently hydrolyse GTP and therefore require guanine nucleotide exchange factors (GEFs) and GTPase-activating proteins (GAPs) to regulate their nucleotide binding status^5,6^. In this way, Rabs act as molecular switches that mediate downstream events by interacting with effector molecules when anchored, in their GTP-bound form, to their target membrane compartment.

Novel, large Rab GTPases have recently been described that, in addition to having the highly conserved C-terminal Rab domain, also contain a coiled-coil domain and distinct N-terminal EF-hand domains that have the ability to bind Ca^2+^. To date only three Ca^2+^-sensing GTPases (Rab44; Rab45 (RASEF) and Rab46 (CRACR2A-L)) have been reported. These Rabs play roles in trafficking events in osteoclasts (Rab44)^7^, cancer cells (Rab45)^8^, endothelial cells (ECs) and immune cells (Rab46)^9,10^. Their ability to sense changes in intracellular Ca^2+^ implies that not only do these proteins have the ability to regulate intracellular trafficking events, but they could provide spatial and temporal regulation in response to signalling events (allowing a Ca^2+^ sensor to be in the right place at the right time) thereby providing a rapid link between intracellular signalling and vesicular transport.

We discovered Rab46 (CRACR2A-L) in ECs^11^ and described its important function in coupling stimuli to the appropriate release of cargo from endothelial-specific storage organelles (Weibel–Palade bodies: WPBs)^9^. In response to histamine, but not thrombin, Rab46 diverts a subpopulation of WPBs, carrying cargo superfluous to a histamine (immune) response, away from the plasma membrane to the microtubule organising centre (MTOC), inhibiting cargo release and thus preventing an all-out thrombotic response. In T-cells, Rab46 is a component of sub-synaptic vesicles and important for activation of the Ca^2+^ and the Jnk signalling pathways upon T-cell receptor stimulation^10^. The expression profile of Rab46 suggests that it will also have importance in a wider cellular context and thereby it is crucial that we understand the molecular machinery underlying its function. Active (GTP-bound) Rab GTPases are localized to their specific membrane compartment^12^ where they recruit various effector proteins that mediate biogenesis, transport, tethering, and fusion of membrane-bound organelles and vesicles^13^. Hence identifying effector proteins will be key in understanding the complete function of Rab46. In ECs we have shown that the retrograde trafficking of WPBs is dependent on the GTP-bound state of Rab46 and is mediated by dynein-dependent movement along microtubules. Indeed, Wang et al. have suggested that Rab46 may be a direct Ca^2+^-dependent dynein adaptor in T-cells^14^. However, we do not know if dynein is an effector protein in ECs considering the dynamic and multi-functional structure of Rab46.

**Figure 1:**
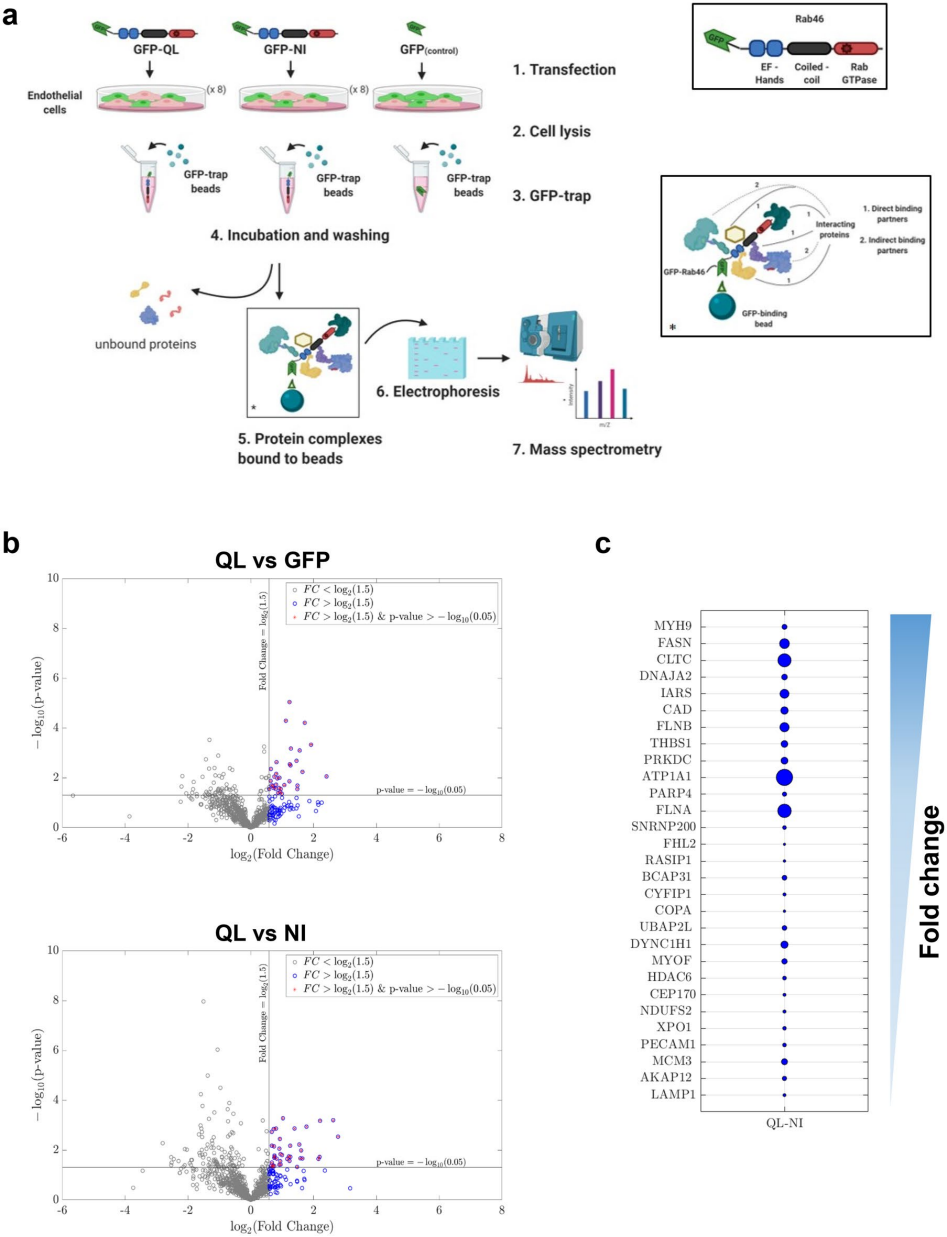
Identification of Rab46 interactome in endothelial cells. (**a**) A schematic showing the workflow for the pull-down experiments demonstrating the major steps for purification of GFP-tagged Rab46 mutants and interacting proteins. ECs transfected with GFP-tagged Rab46 mutants were lysed and incubated with GFP-trap beads. After incubation the beads were sedimented and following several wash steps the protein complex bound to the beads was eluted with appropriate buffer. 10% of the beads were used for SDS-PAGE/Coomassie staining and western blot analysis. Proteins interacting with the bait protein co-purify on the affinity beads and are subsequently identified using mass spectrometry. Drawing created using BioRender.com. (**b**) Volcano plots showing the distribution of the significant proteins that co-precipitated with GFP-tagged Rab46 nucleotide binding active mutant (Q604L) versus GFP control, or versus inactive N658I mutant. Compared values of significant peptides identified from mass spectrometry analysis were plotted according to *p* value (− log_10_ transformed) on the y-axis and fold change (log_2_ transformation) on the x-axis. Significance level is indicated with a horizontal straight black line (*p* value < 0.05) and fold change threshold with vertical black line (fold change ≥ 1.5). Blue data points represent a first sorting filter to highlight proteins with a fold change greater than 1.5 and purple data points highlight proteins also with a significant *p* value. Volcano plot created with MATLAB 2018b using a custom written algorithm. (**c**) Proteins co-precipitate with GFP-Q604L in reference to GFP-N658I, were sorted by fold change (all greater than 1.5, descending values as per arrow) and visualized using a bubble plot where the circle area is proportional to *p* values (bigger circle = lower *p* value). Bubble plot created with MATLAB 2018b (https://www.mathworks.com/matlabcentral/fileexchange/48005-bubbleplot-multidimensional-scatter-plots).

Here, we provide for the first time, a non-biased screen of proteins that interact with the active form (GTP-bound) of Rab46 by using affinity purification and liquid chromatography tandem mass spectrometry (LC–MS/MS: see Fig. 1a). In total, 922 peptides were identified that significantly interacted with Rab46 Q604L or N658I mutants as shown in the appendix provided (Table 1: a resource for further analysis). Many proteins can interact with Rab GTPases such as, GAPS, GEFs, GDIs, effector proteins or proteins involved in translation, protein sorting and processing. Thereby, to identify candidate Rab46 effectors, we ranked peptides pulled down by GTP-locked active Rab46 (Q604L mutant) that displayed a significant > 1.5 fold change over those that interacted with a GDP-locked inactive Rab46 (N658I mutant) or our GFP control. This enrichment generated 29 candidate effectors with GO terms associated with membrane trafficking. Importantly, we validated the interaction of two of these proteins with endogenous Rab46 in ECs. First, we support a role for Rab46 as an adaptor for dynein (which we have already shown is necessary for Rab46-dependent trafficking^9^), and we suggest this direct interaction potentially acts through the Rab46 coiled-coil domain, a domain necessary for interaction in other dynein adaptors^15^. Furthermore, we validate the use of this resource for further investigations by confirming interaction of another highly ranked protein, ATP1α1 with endogenous Rab46.

We propose a first map of novel candidate proteins which may open up new routes in understanding Rab46 mechanism of action in ECs and potentially in a wider cellular context. Characterization of novel key proteins in the Rab46 network could allow the identification of new targets to be exploited as modulators of its physiological function.

## Results

### Identification of Rab46-interacting proteins in endothelial cells

To understand the molecular mechanism underlying Rab46 function in ECs, we sought to identify interacting protein partners. To isolate potential Rab46 effector proteins, we considered the constitutively active GTP-bound mutant (Q604L) compared to the inactive GDP-bound mutant (N658I) for proteomic analysis, as most known Rab effector proteins preferentially interact with the GTP-bound form of the Rab GTPase. We performed affinity chromatography, followed by LC–MS/MS to define proteins that bound to the Q604L and the N658I GFP-Rab46 mutants. GTPase deficiency of both the mutants has been previously validated^10^. Figure 1a illustrates the general workflow for the GFP-trap pull down and protein identification by mass spectrometry. Quantitative data extraction for all identified protein was performed in PeakView and peptides were statistically filtered using false discovery rate (FDR) < 1%.

Pulldown experiments and LC–MS/MS identified a total of 922 unique peptides that significantly co-purified with active Q604L or inactive N658I. The identified peptides are derived from proteins that are potential novel Rab46 interactors and thus, this dataset provides a valuable resource for studying Rab46 biology. However, as many of these proteins could be GAPS, GEFS, GDIs, effector proteins, proteins involved in the synthetic pathway or indeed non-specific binders that would not be localised together in vivo, to extricate proteins for further analysis, we enriched for likely Rab46 effectors by pairwise comparisons. Here, this comparative analyses provides two datasets: (i) QL vs NI, and (ii) QL vs GFP (Tables 1, 2). To identify candidate effectors with increased confidence we firstly considered that true interacting proteins would bind with a higher specificity to GFP-Rab46 mutants than GFP alone, hence we extracted Q604L bound peptides that displayed a significant (*p* value ≤ 0.05) > 1.5-fold change in abundance from peptides that bound GFP (volcano plot Fig. 1b top). We then assumed that effector proteins would display increased binding specificity to the active Q604L form than the inactive N658I mutant, so we applied the same criteria to extract Q604L abundant bound peptides compared to N658I bound peptides (volcano plot Fig. 1b bottom). We identified 29 Q604L enriched proteins (3.5% of total proteins) with significant *p* value compared to N658I peptides and 34 Q604L enriched proteins compared to GFP. Finally, in Fig. 1c (and S1) we ranked these Q604L bound proteins according to their fold change with their respective *p* value represented by bubble size (bigger circle represents smaller p values). Co-purified impurities such as keratin and trypsin were removed from this list, as well as typical nonspecific binders such as heat shock proteins and elongation factors ^16^.

**Figure 2:**
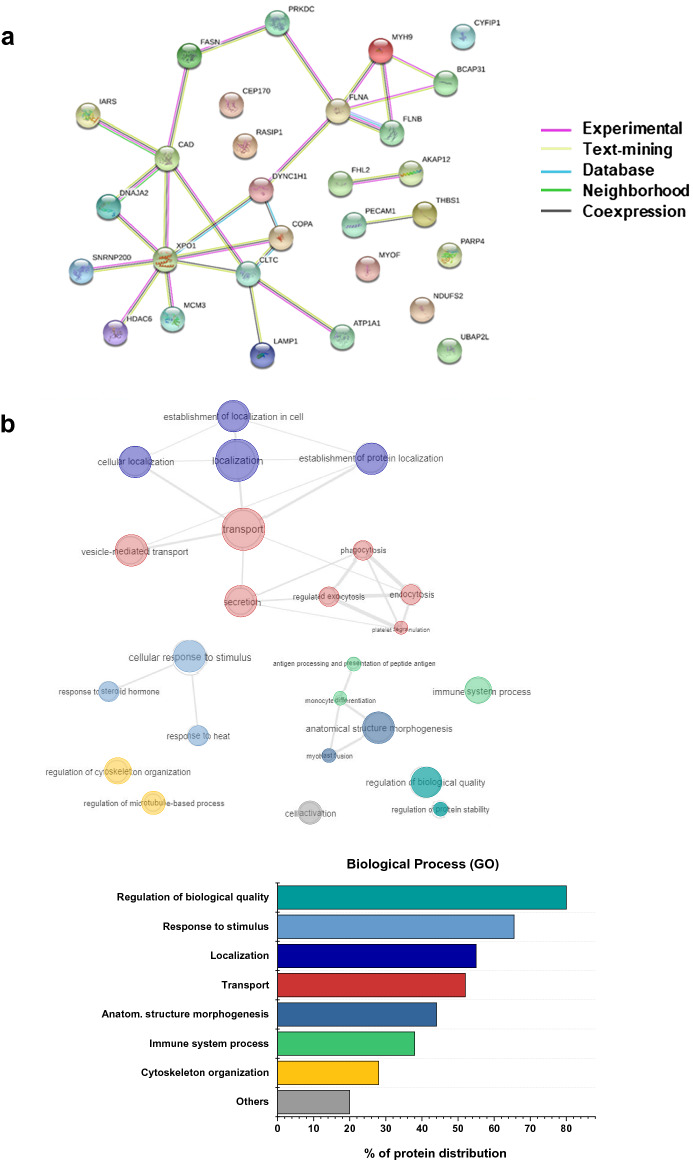
Functional profiling of potentially new Rab46 interacting partners. (**a**) The Search Tool for the Retrieval of Interacting Genes/Proteins database (STRING v10.5) is used to construct the PPI network of 29 proteins identified in the QL vs NI dataset. Network contains 29 nodes, 26 edges (vs 12 expected edges), 1.8 average node degree, 0.58 avg. local clustering coefficient. PPI enrichment *p* value 0.000254. Different colors of the lines represent the types of evidence used in predicting the association. (**b**) GO enrichment analysis elucidating enriched biological process (BP). GO terms identified using dataset imported from STRING and analyzed with REVIGO web server (http://revigo.irb.hr/). Redundant GO terms were excluded for clarity. Results are visualized with REVIGO interactive maps showing enriched BP GO terms. Similar GO terms are color coded. The color matches the bar colors of the bar chart on the right showing percentage of protein distribution among the most represented GO terms identified.

The differentially expressed proteins in Q604L vs N658I (n = 29) were investigated for biological function using STRING database (https://string-db.org/) to establish known and predicted protein interactions (Fig. 2a). The gene ontology (GO) classification system was used to elucidate biological processes, cellular components and molecular functions of target proteins. GO terms were submitted to REVIGO (http://revigo.irb.hr/) and the results visualised using interactive plots (Figs. 2b, S2) to help reveal identified patterns within the data in a comprehensive and biologically meaningful manner. Interestingly, Fig. 2b shows a central node for transport processes with different edges pointing at more specific function such as localization, secretion, vesicle-mediated transport and microtubule-based transport. Therefore, clear clusters emerge within the biological process for regulation of trafficking events. Moreover, analysis revealed highly represented GO cellular component terms from intracellular organelles including cytoplasmic vesicles, secretory granules and endocytic vesicles as well as cytoskeleton components (Fig. S2). GO molecular function terms have also been considered showing many binding function terms, including different subsets, to be the most abundant (Table 3). Altogether the function enrichment analysis showed a high proportion of terms associated with membrane and vesicular trafficking processes which are of particular interest for understanding the role of Rab46 in ECs.

### Rab46 interacts with the dynein/dynactin complex

Among the set of candidate Rab46 effectors identified by enrichment of the proteomic analysis, the dynein heavy chain (DHC) constitutes a well-known motor protein involved in cargo trafficking, therefore most likely to be a relevant physiological interactor for a small Rab GTPase. Our previous findings revealed that histamine evoked Rab46-dependent retrograde trafficking of WPBs to the MTOC^9^. Moreover, we demonstrated the functional consequence of inhibiting dynein, since treatment of HUVECs with ciliobrevin D abolished the histamine evoked retrograde trafficking (see Fig. 5d in^9^). Recently, the identification of Rab46 as a new dynein adaptor protein in immune cells by Wang et al.^14^ advocates DHC as a promising candidate for further investigation.

**Figure 3:**
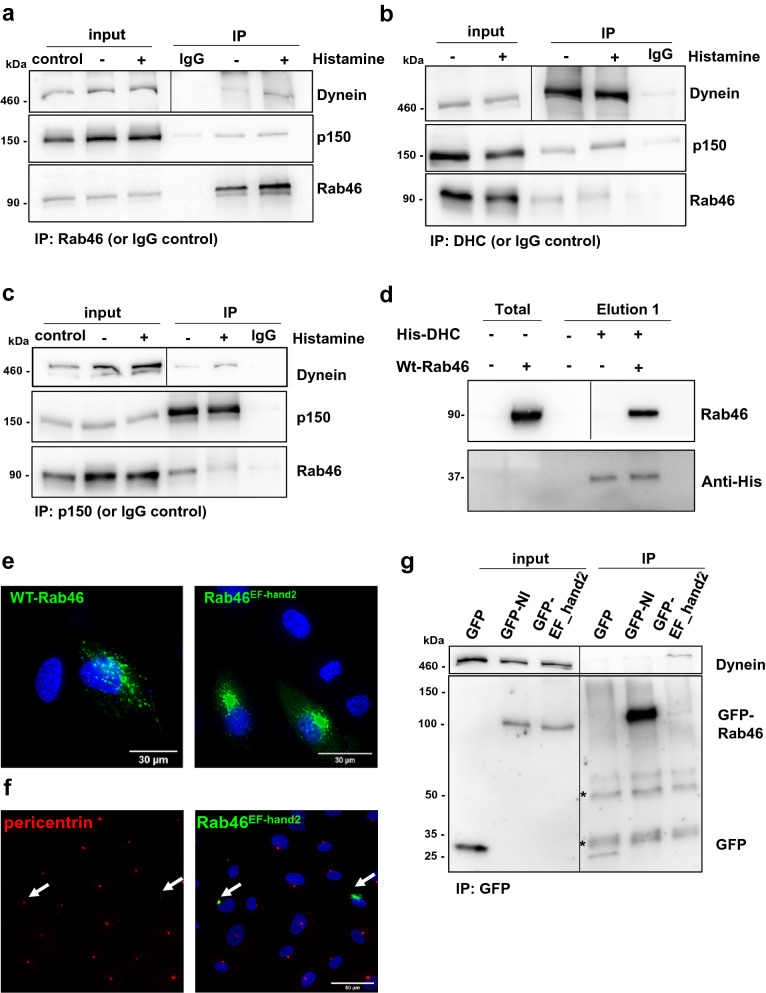
Rab46 interacts with the dynein/dynactin complex in a Ca^2+^ independent manner. a, b and c represent Co-IPs of endogenous Rab46 (a), DHC (b) and p150 (c). The input lanes represent the total amount of the proteins of interest in the lysate prior to IP and indicate loading. The lysates are from histamine, vehicle treated and control cells prior to pull down with either the named antibody or IgG isotype (control sample). (**a**) Immunoprecipitation (IP) of endogenous Rab46 in HUVECs stimulated with 30 μM histamine or vehicle was performed using an anti-Rab46 antibody and Co-IP of DHC and p150, subunit of dynactin, was assessed by immunoblotting. Note the interaction between dynein and Rab46 has been shown previously^9^. Here we show the interaction is part of a complex with p150. (**b**) Reverse Co-IP of endogenous Rab46 and p150 subunit was performed using anti-DHC antibody, Co-IP was assessed by immunoblotting for Rab46 and p150. (**c**) Reverse Co-IP of endogenous Rab46 and DHC was performed using anti-p150 antibody, Co-IP was assessed by immunoblotting. Input represents total lysate before the IP and samples after the IP are denoted as IP. Corresponding IgG were used as negative control. Blots are representative of 3 independent experiments. (**d**) Pull down of human WT-Rab46 overexpressed in Cos7 cells using purified his-tag DHC (His-DHC) bound to Ni^2+^ beads. Non-transfected cells (–) and cells transfected with WT-Rab46 (+) were mixed with (+) or without (–) 10 μg of His-DHC. Magnetic sepharose Ni^2+^ beads were used to pull-down His-tag DHC complexes: the beads were incubated with the mixture of His-DHC + WT-Rab46 (or non-transfected cells as control) or WT-Rab46 only as control to show non-specific binding to the beads. Pull down of Rab46 with his-DHC was assessed by western blot using anti-Rab46 and anti-histidine antibodies. Western blots are representative of 3 independent experiments. (**e**) Representative images of HUVECs expressing WT-Rab46 or Rab46 EF-hand2 mutant (green). Scale bar = 30 μm. (**f**) Deficiency in Ca^2+^ binding shows Rab46 EF-hand2 mutant clustering in the perinuclear area at the MTOC. Pericentrin (red: left image) acts as marker for the MTOC. Arrows depict cells where pericentrin (right and left images) and Rab46 EF-hand2 mutant (green) co-localise at the MTOC in the merged image (right). DAPI (blue) represents nuclei. Scale bar = 50 μm. **g)** Immunoprecipitation of Rab46 binding mutants (GFP-N658I and GFP-EF hand2 mutant) in HUVECs was performed using an anti-GFP antibody and Co-IP of DHC was assessed by immunoblotting for DHC. Input represents total lysate before the IP and samples after the IP are denoted as IP. Empty GFP vector was used as negative control. (*) indicates non-specific bands corresponding to the IgG heavy (50 kDa) and light chains (25 kDa). Note in all blots/IPs the black line delineates a cropped image. Raw images of membranes are available in Fig. S5.

We have previously validated the interaction between endogenous Rab46 and a complex containing DHC in ECs (data shown again in Fig. 3a for context)^9^. If Rab46 is a dynein adaptor then, in a manner similar to other dynein adaptors, Rab46 should also form a complex with dynactin. The largest component of the dynactin complex is p150^Glued^ which binds to dynein and mediates direct association between dynactin and microtubules^17^. Prior to IP the cells were treated with histamine or vehicle to determine if histamine stimulated-evoked trafficking was regulated by GTP binding thereby mimicking the Q604L GTP-bound mutant. Co-immunoprecipitation (Co-IP) using an anti-Rab46 IgG (verified previously in^9^) or IgG negative control resulted in pull down of endogenous Rab46 from histamine and non-histamine treated ECs and demonstrated the interaction between Rab46 and the dynein–dynactin complex via both DHC and p150^Glued^ (Fig. 3a). Reverse immunoprecipitation (IP) also verified complex formation in ECs (Fig. 3b,c). Probing for these proteins simultaneously (p150, Rab46 and DHC) revealed a specific band at the expected molecular weight for endogenous Rab46 (95 kDa) in both the DHC and p150 IP samples (Fig. 3b,c). The inputs (Fig. 3a–c) demonstrate that histamine treatment does not affect the levels of the proteins to be immunoprecipitated in each sample and acts as a loading control. The association of Rab46, DHC and p150 suggested the existence of a Rab46–dynein–dynactin motor complex, in a manner similar to other dynein adaptors. Since Co-IP of dynein by the anti-Rab46 and p150 antibodies occur in the presence and absence of histamine, this suggests that histamine does not affect the GTP-bound state of Rab46 and therefore histamine-evoked Rab46 trafficking is not via GTPase activity.

To indicate the integrity of the Rab46/dynein interaction we sought to determine whether human Rab46 could interact with the dynein complex in a cells that don’t express Rab46. Firstly, we ectopically expressed human Rab46 (hRab46) in Cos-7 (monkey) cells and used a purified recombinant portion of the tail domain of human DHC (His-DYNCH1) as a bait to identify binding partners. These cells do not express hRab46 and therefore present as a suitable system to indicate direct interactions. Western blot analysis (Fig. 3d), revealed the presence of recombinant dynein (37 kDa) in the positive control and in the elution fraction after incubation with lysates containing hRab46. Probing for Rab46 revealed a single band at the expected molecular weight (95 kDa) in the pulled down fraction. No Rab46 was detected in cells not expressing hRab46 and no cross-reactivity was observed in the negative control where WT-Rab46 was incubated with the Ni^2+^ beads but in absence of His-DHC. These results suggest an interaction occurs between hRab46 and hDHC in a non-human model. Furthermore, in vitro assays performed by Wang et al. (see Fig. 1B in *J Cell Biol* **218**, 1619–1633 (2019)) using recombinant full length Rab46 and DHC indicate a direct interaction between the two proteins.

### Rab46/dynein interaction is independent of calcium

Rab46 contains two Ca^2+^ binding motifs (EF-hands) in the N-terminal, the 2nd of which binds Ca^2+^^18^. Wang et al. recently reported that Ca^2+^ binding to the EF-hands was necessary for the interaction between Rab46 and dynein in T-cells^14^. This is surprising because in ECs histamine and thrombin elicit a robust store-operated Ca^2+^ response^9, however^ only histamine evokes activation of Rab46-dependent trafficking, indicating this is not a Ca^2+^-dependent event but something particular to histamine signalling. To evidence this, we have previously demonstrated that chelation of intracellular Ca^2+^ with BAPTA does not inhibit Rab46/ dynein-dependent retrograde trafficking of WPBs to the MTOC^9^ and a mutant of Rab46 that is unable to bind Ca^2+^ (2nd EF-hand mutant: Rab46^EFhand2^) is recruited to WPBs (see^9^ reviewers notes online) and rapidly becomes localised to the MTOC even in the absence of stimulation (see Fig. 3e,f, examples of the cellular distribution of WT-Rab46 and EF-hand2). Indeed, in contrast to T-cells, release of Ca^2+^ from stores had no effect on the distribution of Rab46, unless Rab46 has already clustered at the MTOC, where Ca^2+^ binding to Rab46 is necessary for anterograde transport away from the MTOC. In order to further validate that, in ECs, the Rab46-dynein interaction was independent of Ca^2+^, we heterologously expressed the GFP-tagged Ca^2+^ binding mutant of Rab46, Rab46^EF-hand2^ (Fig. 3g) and compared its ability to pull down dynein to the N658I Rab46 (that we have previously shown fails to pull down dynein^9^). The input shows that these mutants were expressed at similar levels, however, even though the level of Rab46^EF-Hand2^ in the IP fraction is lower than the inactive GFP-N658I mutant, a distinct dynein band, at the correct molecular weight (460 kDa) was observed in this fraction as opposed to the N658I mutant or GFP control. These results support our previous cellular imaging studies^9^ that suggests the interaction between Rab46 and dynein is Ca^2+^-independent in ECs.

Altogether these observations in ECs support the evidence given by Wang et al., which describe Rab46 as a new dynein adaptor in T-cells. However, together with our previous data, this indicates complex formation between Rab46 and dynein–dynactin is insensitive to changes in intracellular Ca^2+^ in ECs.

### Identification of binding domains that enable Rab46/dynein interaction

**Figure 4:**
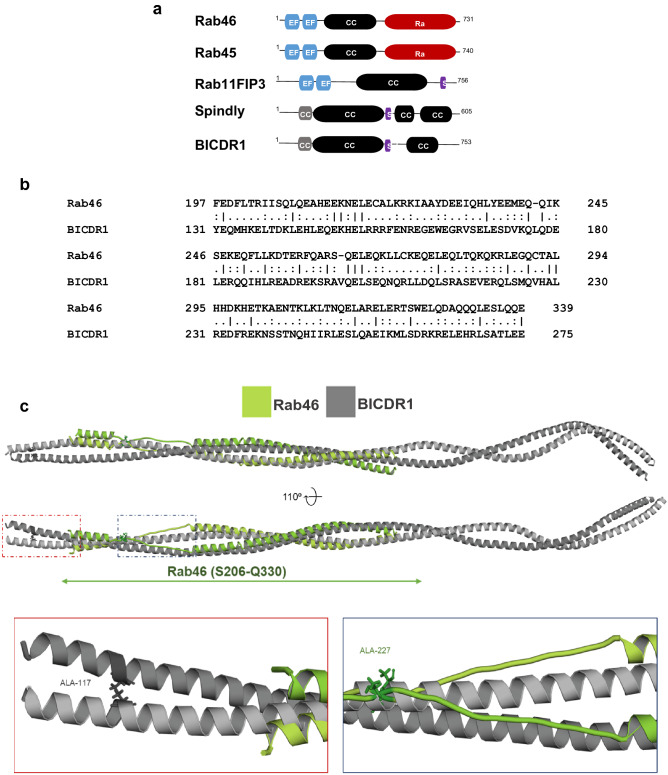
Rab46 is a novel dynein/dynactin adaptor. (**a**) Domain organization of structural related Rab GTPases (Rab46 and Rab45) compared to Rab11FIP3, Spindly and BICDR1 (dynein activators and adaptors). EF-hand domains (blue): calcium binding sites. Coiled-coil domain (CC: black): protein interactions sites. Rab (red): GTP/GDP binding site. CC1 (grey): coiled-coil segment shared by some dynein binding proteins. Spindly motif (S: purple): conserved features of some dynein activators. (**b**) Amino acid sequence alignment exposed from full length pairwise analysis of BICDR1 (CC1) and Rab46 using EMBOSS Matcher. Alignment shows 20% identity and 50.3% similarity between the first coiled-coil segment of the dynein adaptor BICDR1 (CC1 box) and Rab46 coiled-coil domain (top). (**c**) Homology model of Rab46 coiled-coil domain. Top: Rab46 coiled-coil structure (green) computed by the SWISS-MODEL server and superimposed on CryoEM structure of BICDR1 (grey) using PyMOL. Bottom: expanded boxes showing detailed views of the position of a conserved alanine residues in BICDR1 (left) and Rab46 (right) coiled-coil domains.

Rab46 is an unconventional Rab that contains a pair of EF-hands, a coiled-coil domain and a Rab GTPase domain (Fig. 4a). The domain architecture resembles some well-known dynein adaptors (Fig. 4a). A common feature of dynein adaptor proteins is the presence of long coiled-coil domains that are important for interaction with dynein. However, the sequence similarity between these coiled-coil domains is low^19,20^ a feature that allows diverse adaptors to differentially engage with the dynein/dynactin complex and thereby elicit varying trafficking kinetics. The dynein adaptor BICDR1 contains three coiled regions and it interacts with dynein via the first^21^. Comparison of the full amino acid sequence of Rab46 and BICDR1 by pairwise sequence alignment, revealed a single domain that was similar between the two. The BICDR1 first coiled-coil box (CC1) and Rab46 coiled-coil domain displayed 20% identity and 50.3% similarity (Fig. 4b). Moreover, a conserved alanine residue responsible for dynein binding in the BICDR1 sequence was identified in the Rab46 sequence (A227 highlighted in red Fig. 4c), an alanine that is conserved in other dynein adaptors including BICD2, BICDR2, Spindly and HAP1^15,22^.

Alignment of the computed Rab46 model to all structures in the PDB library identified one of the chains of BICDR1 dynein adaptor (6F1TX) as a top structural homolog of Rab46 coiled-coil (TM-score = 0.672). The alignment of Rab46 coiled-coil and BICDR1 structures demonstrated structural similarity between the proteins (reported RMSD = 4.49 Å) (Fig. 4c). Although RMSD value of less than 3 Å would typically be expected for homologous proteins^23^ there are no high-resolution structures of dynein adaptor proteins currently available to be used as modelling templates. The positions of conserved alanine residues in BICDR1 and Rab46 are shown in the expanded boxes in Fig. 4c.

These preliminary results suggest Rab46 is a dynein adaptor that may interact with the dynein/dynactin complex through its coiled-coil domain.

### Rab46 interacts with the Na^2+^/K^+^ ATPase subunit alpha 1

Na^2+^/ K^+^ ATPase subunit α1 (ATP1α1) was identified as another potential Rab46 effector protein in our enriched dataset. To date, the role of ATP1α1 in membrane trafficking has not been investigated. However, recently ATP1α1 has been identified as a new effector for Rab27a^24^. Therefore, we decided to further investigate the potential interaction between Rab46 and the ATP1α1.

**Figure 5:**
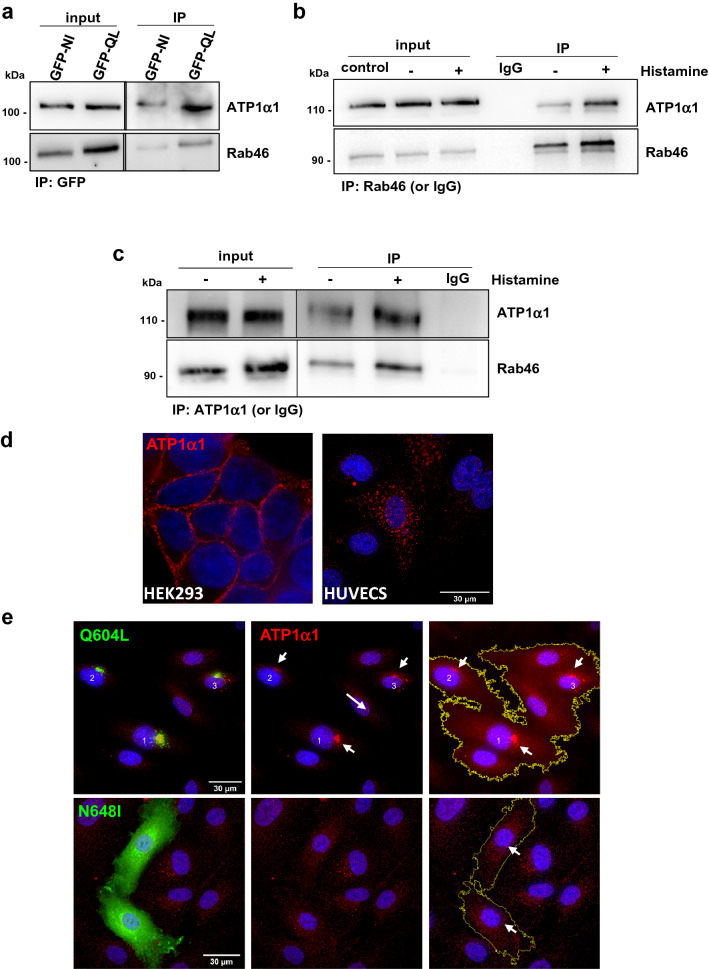
Rab46 interacts with the Na^2+^/ K^+^ ATPase subunit α1. (**a**) IP of Rab46 nucleotide binding mutants to confirm proteomic analysis. GFP-tagged active and inactive form of Rab46 (Q604L and N658I respectively) were overexpressed in HUVECs and IP performed using an anti-GFP antibody. Input represents lysate before IP and IP denotes samples after IP. Western blotting using anti-GFP and anti-ATP1α1 antibodies shows that ATP1α1 Co-IPs with the active form of Rab46 (Q604L). (**b**,**c**) Endogenous Rab46 interacts with ATP1α1. IP of endogenous Rab46 in HUVECs stimulated with 30 μM histamine or vehicle control was performed using an anti-Rab46 antibody and Co-IP of ATP1α1 was assessed by immunoblotting (b). Reverse Co-IP of endogenous Rab46 performed using anti-ATP1α1 antibody, Co-IP was assessed by immunoblotting for Rab46. Input represents total lysate before the IP and samples after the IP are denoted as IP (c). Anti-rabbit and anti-mouse IgG were respectively used as negative controls. Blots are representative of 3 independent experiments. (**d**) Representative immunofluorescent images of ATP1α1 (red) localization in HEK293 (left) and HUVECs (right). DAPI (blue) shows nuclei. Scale bar = 30 µm. (**e**) Representative images of HUVECs expressing Rab46 nucleotide bindings mutants (green): Q604L (top), N658I (bottom) and ATP1α1 (red) expression. DAPI (blue) shows nuclei. Scale bars = 30 µm. The arrows show the distribution of ATP1α1 in cells that are transfected with mutant Rab46. Immunostaining and imaging were performed on three biological repeats and included a minimum of three images per condition containing multiple cells. Note in all blots/ IPs the black line delineates a cropped image. Raw images of membranes are available in Fig. S6.

Validation of the interaction between Rab46 and ATP1α1 was performed using IP and western blot analysis in lysates from cells expressing GFP-tagged constitutively active (Q604L) Rab46. A band size of 110 kDa was detected in the IP fractions and confirmed as ATP1α1. Specificity of the antibody has been validated by siRNA experiments (Fig. S3). Notably, the ATP1α1 interacts with the active GTP-bound Rab46 and the inactive Rab46 (Fig. 5a).

To validate this result for the native protein, endogenous Rab46 IP was performed and ATP1α1 Co-IP assessed by immunoblotting. As histamine evokes trafficking of Rab46 by an as yet unknown signalling pathway, ECs were treated with histamine or vehicle prior to IP to see if histamine mediated the interaction between Rab46 and ATP1α1. Co-IP demonstrated association of endogenous Rab46 with ATP1α1 (Fig. 5b) and a band is visible for ATP1α1 in the histamine and non-histamine-treated samples. This interaction was validated with a reverse Co-IP using an antibody against the novel target (ATP1α1) and immunoblotting for the original “bait” (Rab46). Similarly, the reverse Co-IP revealed the presence of Rab46 in both the ATP1α1 IP fractions (Fig. 5c). The specificity of this interaction is supported by the use of control IgG as negative control showing no interaction between the IgG and the target proteins.

These results suggest that ATP1α1 is a Rab46-interacting protein and histamine stimulation is not necessary for this interaction.

### Na^2+^/K^+^ ATPase subunit alpha 1 expression in endothelial cells

To date, the expression and the role of ATP1α1 in ECs has not been investigated. ATP1α1 was depleted in ECs by targeted siRNA and the antibody specificity validated with western blot (Fig. S3). A single band at the known molecular weight (110 kDa) was observed in the mock and control siRNA transfected cells whereas a reduced band intensity was observed in ATP1α1 siRNA transfected cells (Fig. S3). This specificity encouraged the use of this antibody for immunofluorescence imaging studies. Using high resolution imaging, we observed an intracellular vesicular-like localization of ATP1α1 in ECs (Fig. 5d), in contrast to HEK293 cells which express ATP1α1 at the plasma membrane. Moreover, the GTPase activity of Rab46 affected the intracellular localization of ATP1α1 where, ATP1α1 co-localised with the active form of Rab46 (Q604L) in the perinuclear area of ECs (Figs. 5e and S4). Moreover, ATP1α1 displayed a cytosolic granular distribution in cells expressing the inactive N658I mutant of Rab46.

## Discussion

Rab46 is a novel Ca^2+^-sensing Rab GTPase which was first discovered in ECs by Wilson et al.^11^. The contribution of Rab46 to the function of the endothelium^9^ and to T-cells^10^ has recently been defined, however, the interacting proteins that regulate this function have yet to be described. In the present study we have taken an unbiased approach to identify Rab46 effector proteins by deploying label-free proteomics to generate a comprehensive list of candidate Rab46 effectors in ECs. In addition, further analyses of the candidate proteins provided a high confidence prediction of novel interactors. We have validated our findings by exploring two putative interactors; dynein and ATP1α1. Thus, here we provide a useful resource for investigating Rab46 protein–protein interactions which could serve to identify the molecular mechanisms that regulate this novel Rab GTPase.

Analysis of the 29 statistically significant enriched proteins and functional enrichment studies in our proteome dataset support the involvement of Rab46 signalling in intracellular trafficking events. Indeed, integration of functional information and topological information highlighted key proteins involved in relevant biological mechanisms. For instance, some proteins such as XPO1, DHC, FLNA, MYO9 and CYFIP1 which have already been characterized as effector proteins have been highlighted in the Rab46 network, together with proteins like COPA, CLTC, LAMP1 and ATP1α1 which appear to be enriched in transport and localization processes. Because protein function and location are often tightly linked, it was also interesting to observe that several of the enriched proteins are part of intracellular components such as organelles, vesicles and cytoskeleton parts. Therefore, the three ontology domains covered by the analysis strongly place Rab46, which is a non-conventional Rab GTPase, in the context of intracellular trafficking where direct or indirect binding with the proteins identified could lead to the regulation of different pathways in the endothelium.

In this study, we provide evidence that Rab46 interacts with the dynein/dynactin motor complex in ECs, corroborating the previously published evidence by Wang et al. proposing Rab46 (CRACR2A-L) as a new dynein adaptor in T-cells^14^. However, in contrast to Wang et al. here, we have shown that in lysates from ECs a Rab46 EF-hand mutant that cannot bind Ca^2+^ can interact with dynein, indicating this interaction is independent of Ca^2+^. This supports our previous studies in ECs where we have shown extensive evidence that histamine evoked trafficking of Rab46 to the MTOC is dependent on dynein but independent of Ca^2+^ (J. Cell Biol.^9^).There are several explanations of this discrepancy, firstly this difference might reflect distinction in the physiology and/or the accessory protein expression between the two cell types (ECs and T-cells). For instance, upon T-cell activation, the MTOC and Rab46 both traffic to the immunological synapse^14^ whereas in ECs there is a retrograde movement of Rab46 along the microtubules towards MTOC upon stimulation^9^. In addition, whilst ECs only express Rab46^11^, T-cells also express the short non-Rab isoform (CRACR2A-S) of the gene (*EFCAB4B*) that lacks the Rab domain^18^. Both Rab46 and CRACR2A-S are necessary for regulating store-operated Ca^2+^ entry (SOCE) in T-cells but we found no function of Rab46 in SOCE in ECs^11^, suggesting an interplay between these two isoforms. It is also interesting to note that the non-Rab isoform (CRACR2A-S) contains the EF-hand and coiled-coil domains of Rab46 but does not interact with dynein. Secondly, Rab46 contains two EF-hand motifs of which only the 2^nd^ motif binds to Ca^2+^^18,29^. Functional pairs of EF-hands are necessary for the correct folding of proteins and the crystal structure of Rab46 EF-hands display a canonical fold, consisting of incoming helix (helices α1 and α3), loop and exiting helix (helices α2 and α4)^29^. The recombinant protein expressed in bacteria used by Wang et al. for the *in vitro* interaction assay had a double EF-hand mutation and there was no data regarding the correct folding of this protein. In addition, they showed recombinant WT-Rab46 pulled down either dynein or dynactin in the absence of Ca^2+^ and in the presence of EGTA. However, we should also consider that the 1st EF-hand may play important roles in setting the affinity of the 2nd EF-hand so that the 2nd EF-hand can respond to changes in intracellular Ca^2+^ upon signalling events, this can only be determined by rigorous structural and biophysical analysis.

Association of Rab proteins with motor complexes allow directional movement of various vesicular cargos along the microtubule cytoskeleton. Recent work has provided new insights into the regulation of cytoplasmic dynein by adaptor proteins which link dynein to cargo^25–27^. Structural studies have also described some common feature between dynein adaptors demonstrating that the coiled-coil domains play crucial roles in dictating the interaction with dynein^21,28^. Given the Rab46 similarity with other dynein adaptors, such as BICDR1, sequence alignment predicted a potential conserved binding domain within Rab46 coiled-coil domain which may be necessary for the protein–protein interaction. Domains outside of the coiled-coil region in adaptor proteins could facilitate or regulate their interactions with dynein/dynactin. Moreover, our homology modelling suggests some structural similarity between Rab46 and other dynein adaptors, indicating that manipulation of the conserved residues could further elucidate the role of Rab46 as a dynein adaptor. In addition, we have proposed (see^9^) that, in ECs, Ca^2+^ binding to Rab46 is necessary for release from microtubules at the MTOC. It would therefore also be interesting to observe how Ca^2+^ binding to the EF-hands affects the structure of Rab46 and how this impacts on binding to the dynein complex. Interestingly, Lee et al.^29^ recently published a crystal structure of Rab46 EF-hands in complex with a dynein fragment (LIC1_433–458_). Whilst the EF-hand structure is good in terms of refinement stats and the density map for Ca^2+^ is clear, the use of a short helical fragment (LIC1) could create artifacts. It would also be surprising that an EF-hand played a direct role in protein–protein interactions as these are known Ca^2+^ sensors that, upon changes in intracellular [Ca^2+^], bind Ca^2+^ which then elicits rearrangement of the surrounding helices, resulting in the exposure of the hydrophobic surfaces used to recruit target proteins. However, there are domains within Rab46 with similarity to the LIC1 fragment which is suggestive of intramolecular interactions.

Here, we have also identified and validated a second Rab46 interacting protein, the alpha 1 subunit of the Na^2+^/K^+^ ATPase. It has been reported that ATP1α1 is expressed in a tissue- and developmental-specific manner and is primarily localised at the plasma membrane of cells such as neurons and kidney cells^30,31^. For the first time we reported the specific localization of ATP1α1 in resting ECs. In contrast to other cell types, we have shown that ECs present a more intracellular vesicle-like localization. Following the activation of Rab46 which leads to its perinuclear clustering, we have also shown a redistribution of ATP1α1, which clusters and co-localizes at the perinuclear area with Rab46. Translocation of membrane proteins and specific trafficking events sometimes plays an important role in their related biological function, understanding the location of ATP1α1 associated with Rab46 may be important in further defining the critical role of this novel GTPase in ECs. Interestingly, recent studies indicate that ATP1α1 also functions in activating signalling cascades independently of its ion‐transport role. It is proposed that a separate pool of non‐pumping Na^+^/K^+^ ATPase’s mediate this non-canonical function depending on its interactions with various proteins including protein and lipid kinases, membrane transporters, channels, and cellular receptors^32^. Recently, for the first time, a study from Booth et al.^24^ identified a new role for the ATP1α1 as a novel Rab27a interacting protein in melanocytes where several lines of evidence strongly support an essential role for ATP1α1 in the targeting of Rab27a to melanosomes. Future studies will aim to depict whether ATP1α1 is necessary for targeting Rab46 to WPBs, in addition to regulation of WPB trafficking in ECs. Further studies are required to characterize this interaction as it should also be noted that this in vitro pull-down method detects both direct and indirect interactions.

Here, we have identified effector proteins by using a constitutively active (GTP-bound) Rab46. Differences in experimental design and tissue choice might influence our interaction network. Therefore, the choice of the biological system and the method for quantification will typically depend on the biological question of interest. For instance, we observed steric hindrance of the EF-hand by GFP-tag at the N-terminus of Rab46^9^ therefore our approach here would not be suitable for investigating Ca^2+^-dependent proteins and events (which we suggest occur after effector engagement). Quantitative proteomics such as SILAC analysis and dynamic approaches such as proximity-dependent biotin identification (BioID) and new techniques including proximity-dependent ascorbic acid peroxidase labelling (APEX) will provide additional information allowing spatiotemporal resolution of the interactions, although these too have their limitations.

In conclusion, our study presents the first screen for Rab46 protein interaction partners in ECs with many new potential binding partners presented for further evaluation. Following up on the biological significance of novel interactors is beyond the scope of this paper. However, we point at some interesting observations that might be addressed in future studies. Finally, we lay out a rough map of Rab46 effectors that can contribute to regulation of trafficking events, providing a rich resource for the community that has so far been lacking.

## Material and methods

### Cell culture

Pooled Human Umbilical Vein Endothelial Cells (HUVECs) (Lonza Inc, USA or Promocell, UK), grown in endothelial basal cell medium 2 supplemented with EGM-2 Singlequot supplements (Lonza Inc, USA). HUVECs maintained at 37 °C in a humidified atmosphere of 5% CO_2_ and used between passages 1–5. Human Embryonic Kidney 293 cells (HEK293T—Invitrogen, UK) were grown in Dulbecco's modified Eagle medium (DMEM—Invitrogen, Paisley, UK) containing D-glucose, L-glutamine, and pyruvate (Life Technologies, UK) supplemented with 10% Fetal Bovine Serum (FBS—Sigma‐Aldrich), 100 U/m penicillin, and 100 mg/ml streptomycin (Sigma‐Aldrich). Cos-7 cells were obtained from the American Type Culture Collection (ATCC) and maintained in DMEM supplemented with 10% FBS and 100 U/ml penicillin + 100 mg/ml streptomycin. Cells were maintained at 37 °C and 5% CO_2_ in a humidified incubator and used between passage 2 and 20.

### cDNA constructs and Rab46 mutagenesis

The eGFP-C1 plasmid (kanamycin resistant; Clontech; 4731 bp) was used to generate N-terminal GFP-tagged Rab46 mutants. Various mutants of Rab46 were generated by PCR amplification (pHusion DNA polymerase) and site-directed mutagenesis using primers as described in the supplementary table in^9^.

### cDNA transfection

HUVECs were plated either into Ibidi μ-slide 8-well (7*10^4^ cells/ml) or into a 10 cm Petri dish (10*10^5^ cells/ml) and after 24 h they were transfected using Lipofectamine 2000 (Thermo Fisher Scientific). A 3:1 ratio between Lipofectamine and cDNA (100 ng and 6 μg respectively) was used. 1 h after transfection the medium was removed and fresh cell culture medium added. Experiments were performed 24 h post-transfection. COS-7 cells were plated into a 10 cm Petri dish (2*10^6^ cells/petri dish) and transfected after 24 h as described above.

### siRNA transfection

Control siRNA (D-001810-01-05), used as a negative control, and On-TARGET plus SMART pools for ATP1α1 (L-006111-00-0005) were purchased from Dharmacon (Thermo Scientific). HUVECs at 80–90% confluence were used for transfection, which was performed using a 1:3 ratio of 100 nmol/L siRNAs with Lipofectamine 2000 reagent (Invitrogen) diluted in OptiMEM (Gibco) as per the manufacturers’ instructions. Cells were incubated with the transfection solution for 5 h before medium change. Knockdown was assessed via western blotting. Experiments were performed at 72 h after transfection.

### Western blotting

Cells were harvested with NP-40 lysis buffer (ThermoFisher Scientific, UK) with protease and phosphatase inhibitors cocktails (Sigma‐Aldrich). Samples were loaded on 7.5% or 4–20% gels and resolved by electrophoresis. Proteins were transferred to PVDF membranes using Mini Trans-Blot Cell (BioRad). Membranes incubated for 1 h in blocking solution consisting of 5% w/v milk diluted in TBS-T 145 mM NaCl, 20 mM Tris-base, pH 7.4, 0.5% Tween-20 and labelled with primary antibody overnight at 4 °C for DHC (Proteintech—1:1000), Rab46 (CRACR2A: Proteintech 1:800), p150^Glued^ (BD Biosciences—1:1000), ATP1α1 (Santa Cruz—1:500), GFP (ThermoFisher—1:1000) and histidine (BioRad—1:1000). Immunoblots visualised using HRP-conjugated donkey anti-mouse, anti-rabbit secondary antibodies (Jackson ImmunoResearch—1:10,000) and SuperSignal Femto (Pierce).

### Immunoprecipitation

HUVECs plated in 10 cm Petri dishes were starved in serum-free M199 plus 10 mM HEPES medium (Gibco) for 1 h before treatments. Histamine (Sigma‐Aldrich) was used at 30 µM. After treatments cells were quickly washed with PBS and before harvesting they were cross-linked with 1% PFA and then harvested with 250 µl lysis buffer (NP-40). The lysate was quantified and 0.5 mg of total lysate was incubated with 1 µg of antibody or control IgG for 4 h at 4 °C rotating. This mixture was then added to 30 µl of pre-equilibrated Protein G sepharose beads (GE Healthcare Life Science) and incubated under continuous agitation. Beads were washed thoroughly before elution of the bound proteins in 20 µl 4X sample buffer solution (200 mM Tris pH 6.8, 8% SDS, 40% glycerol, 8% mercaptoethanol, 0.1% bromophenol blue) and boiled at 95 °C for 5 min. The elution fraction was loaded onto SDS-PAGE gel and the amount of Rab46, dynein heavy chain, dynactin, GFP and ATP1α1 detected by western blot. All IPs were performed with 3 biological repeats.

### Pull down assay

#### GFP trap

HUVECs plated in 10 cm Petri-dishes were transfected with the appropriate GFP plasmids for 24 h. Cells were washed with cold PBS and lysed with 250 μl NP-40 lysis buffer. Lysates were left on ice for 20 min and then centrifuged at 12000 g for 10 min at 4 °C. The supernatant was then collected and the protein content was quantified using a Bio-Rad assay. 25 μl GFP-Trap bead 50% slurry (Chromotek, Planegg-Martinsried, Germany) was used and all wash steps were performed with washing buffer containing 10 mM Tris/Cl pH 7.5, 150 mM NaCl and 0.5 mM EDTA. GFP-Trap beads were washed 3× with dilution buffer prior to addition to cell lysate. Beads were incubated with cell lysate at 4 °C for 2 h following another wash step (×3). To elute the proteins off the beads 40 μl sample buffer (200 mM Tris pH 6.8, 8% SDS, 40% glycerol, 8% mercaptoethanol, 0.1% bromophenol blue) was added and samples were boiled at 95 °C for 5 min. Western blotting was used for analysis.

#### His-tag

Cos-7 cells were plated in a 10 cm Petri dish and transfected with WT-Rab46 cDNA. 24 h after transfection cells were washed with ice cold PBS and lysed with 400 μl EDTA-free lysis buffer (BOSTER, Pleasanton, CA, USA) plus protease/phosphates inhibitor cocktail EDTA-free 100x (ThermoFisher). Cell lysate was left on ice for 20 min and then centrifuged at 4 °C 12,000*g* for 10 min. Supernatant was collected and protein quantified. 20 μg of total lysate was used to incubate with pre-equilibrated beads. 100 μl of His Mag Sepharose Ni beads (GE Healthcare) were equilibrated with equilibration buffer containing 20 mM sodium phosphate, 500 mM NaCl, and 20 mM imidazole. Immediately after equilibration, total lysate was added and incubated for 1 h at 4 °C, rotating. The pre-cleared lysate was collected and the beads discarded. At this point, 10 μg of recombinant his-tagged dynein heavy chain (DYNC1H1-CloudClone) were mixed with the cleared lysate and incubated for 1 h. After incubation, the mixture containing the complex (WT-Rab46 + His_6_DYNC1H1), was added to 100 μl of pre-equilibrated His Mag Sepharose Ni beads and incubated for 1 h at 4 °C, rotating. At this point the supernatant was discarded and a linear gradient of imidazole (up to 90 mM) was applied to the beads to reduce unspecific binding. Following these washing steps, elution was performed with elution buffer containing 20 mM sodium phosphate, 500 mM NaCl, and 500 mM imidazole. The eluted samples were collected and analysed by western blot.

### Sample preparation for mass spectrometry

Pulldown eluates were processed using the FASP procedure^33^. Samples were loaded on Vivacon 500, 30 k MWCO HY filter vials (Sartorius Stedim Biotech, VN01H22) and centrifuged to concentrate the proteins on the filter. The samples were washed using FASP1 by centrifugation at 7,000 g. The filters were then washed by centrifugation using FASP 2 (100 mM Tris/HCl pH 8.5) ready for trypsin digestion. The samples were then reduced using 50 mM fresh IAA in FASP 2 in the dark for 30 min. Excess buffer was removed by centrifugation and buffer exchanged into FASP 3 (100 mM TEAB—triethyl ammonium bicarbonate). Trypsin was dissolved in FASP 3 to give a 1:200 enzyme to protein ratio and added in a volume of at least 125 µL for overnight digestion (16 h at 30 °C). This was repeated with fresh trypsin for a 5 h incubation. Filters were spun down using 0.5 M NaCl and 150 µl 10% TFA added to reduce the pH. A standard desalting procedure was used^34^. After desalting, the samples were dried in a speed vac.

### LC–MS/MS and peptide identification

Desalted tryptic peptides were dissolved in 5% acetonitrile (ACN), 0.05% TFA and separated on liquid chromatograph Eksigent Ekspert nano LC 400 (SCIEX, Dublin, CA, USA). Liquid chromatography was online connected to Triple-TOF 5600 + mass spectrometer (SCIEX, Toronto, Canada). Samples were pre-concentrated on a cartridge trap column (300 μm i.d. × 5 mm) packed with C18 PepMap100 sorbent with 5 μm particle size (ThermoFisher Scientific, MA, USA) using a mobile phase composed from 0.05% trifluoroacetic acid (TFA) in 2% acetonitrile (ACN). Pre-concentrated peptides were separated on a capillary analytical column (75 μm i.d. × 500 mm) packed with C18 PepMap100 sorbent, 2 μm particle size (ThermoFisher Scientific, MA, USA). Mobile phase A composed of 0.1% (v/v) formic acid (FA) in water while mobile phase B composed of 0.1% (v/v) FA in ACN. Analytical gradient started from 2% B, the proportion of mobile phase B increased linearly up to 40% B in 70 min, flow was 300 nl/min. The analytes were ionized in nano-electrospray ion source, where temperature and flow of drying gas was set to 150 °C and 12 psi. Voltage at the capillary emitter was 2.65 kV.

SWATH data acquisition was done in high sensitivity mode and precursor range was set from 400 up to 1200 Da. It was divided to 67 precursor SWATH windows with 12 Da width and 1 Da overlap. Cycle time was 3.5 s.

Pooled spectral library sample was measured in information dependent mode (IDA). Precursor range was set from 400 up to 1250 Da in MS mode and from 200 up to 1600 Da in MS/MS mode. Cycle time was set to 2.3 s and during each cycle top 20 the most intensive precursor ions were fragmented. Precursor exclusion time was set to 12 s. IDA data were searched against human database in ProteinPilot 4.5 (AB-SCIEX, Canada).

Quantitative data extraction for all identified protein was performed in PeakView 1.2.0.3. The quantitative data were extracted for proteins using FDR < 1%. Quantitative data were extracted using a method with 8 min extraction window.

### Ranking of Rab46 candidate effectors

Extracted data were analysed in MarkerView 1.2.1.1. Pairwise changes in protein levels of: (i) QL vs GFP or (ii) QL vs NI (raw data Tables 1, 2) across samples were determined using t-test. These comparisons (as described below) use fold change values which are calculated by comparing the peak areas and *p* values for significance between the technical and biological triplicates. QL vs NI comparisons were used to identify potential effector proteins and ranked according to fold change (1.5 fold change cut-off) of and *p* values < 0.05. Volcano plots used to display statistical significance (*p* value) versus magnitude of change (fold change) of Rab46 (Q604L) proteomic data were generated with MATLAB 2018b. Enriched peptides which met the set criteria were ranked by fold change and visualized with bubble blot to also show the significance of each protein. Bubble size represents *p* values (− log_10_ *p* values), larger bubbles equal more significant *p* values. Interaction networks and Gene Ontology (GO) enrichment analysis of identified proteins was performed with STRING (http://string-db.org/) using default setting. The enriched GO terms were visualized using the interactive plot with REVIGO (http://revigo.irb.hr/) which was used to remove redundant GO terms and produce graphs highlighting the similarity between the terms. Highly similar GO terms are linked by edges in the plot, where the line width indicates the degree of similarity. The initial placement of the nodes is determined by a ‘force-directed’ layout algorithm that aims to keep the more similar nodes closer together^35^. Origin(Pro), "Version 2019b" OriginLab Corporation, Northampton, MA, USA software was used for bar chart presentation.

### Immunocytochemistry

Cells seeded 8 × 10^4^ cells/ml into Ibidi µ-slide 8 well were grown for 48 h. Cells were fixed with 4% PFA for 10 min, washed with PBS and permeabilised with 0.1% Triton-X solution. Primary antibodies against Rab46 (Proteintech, 1:100) and ATP1a (SantaCruz, 1:100) or pericentrin (Abcam, 1:100) were added to the cells for 1 h at room temperature. Next, a fluorescently labelled appropriate species secondary antibodies was used for 30 min (anti-mouse Alexa 594, anti-rabbit Alexa 488 Jackson ImmunoResearch, 1:300). Cells briefly incubated in Hoechst before being mounted with Ibidi mounting medium. 3 biological repeats were performed per sample.

### DeltaVision wide-field deconvolution microscopy

Cells visualized on an Olympus IX-70 inverted microscope using 40×/1.35 oil objectives supported by a DeltaVision deconvolution system (Applied Precision LLC) with SoftWorx image acquisition and analysis software.10 focal planes at 0.2 µm per z-stack were taken using a Roper CoolSNAP HQ CCD camera. Iterative deconvolution (5×) was performed on z stacks using the proprietary algorithm. The filter sets used were DAPI, FITC, and TRITC. All imaging performed at room temperature. All the images were processed and analysed with Fiji ImageJ^36^. A minimum of 3 images were taken per biological repeat.

### Homology modelling

Prior to modelling pairwise alignment of the full amino acid sequences of Rab46 and BICDR1 was undertaken using EMBOSS Matcher https://www.ebi.ac.uk/Tools/psa/emboss_matcher/. The coiled-coil domain of Rab46 (L201-R373) was modelled as a monomer using I-TASSER server^37^ for protein structure and function prediction (https://zhanglab.ccmb.med.umich.edu/I-TASSER/). Alignment of the computed model to all structures in the PDB library was performed using TM-align tool (https://zhanglab.ccmb.med.umich.edu/TM-align/). A homology model of Rab46 coiled-coil domain dimer was computed using SWISS-MODEL server^38^ (https://swissmodel.expasy.org/). Since sequence identity between coiled-coil domains of dynein adaptors is typically low, the CryoEM structure of mouse BICDR1^21^ (PDB ID 6F1TX and 6F1Tx) was used for template-based protein structure modelling in order to obtain a parallel homodimeric model of Rab46 coiled-coil. The structures of BICDR1 (105–392) and Rab46 (206–330) coiled-coils were pre-processed and energy-minimised^39^ using Protein Preparation Wizard (Maestro software, Release 2020-1, Glide, Schrödinger, LLC, New York, NY, 2020). The steric clashes generated during modelling were removed using Chiron web server^40^. Superimposition of the protein structures and RMSD calculations were performed using PyMOL (The PyMOL Molecular Graphics System, Version 2.0 Schrödinger, LLC.).

## Supplementary Information

Supplementary Information 1.

Supplementary Information 2.

Supplementary Information 3.

Supplementary Information 4.

## Data Availability

The datasets generated or analysed during this current study are included in the supplementary information file.

## Abbreviations

LC–MS/MS: Liquid chromatography tandem mass spectrometry
GEFs: Guanine nucleotide exchange factors,
GAPs: GTPase activating proteins
WPBs: Weibel–Palade bodies
ECs: Endothelial cells
MTOC: Microtubule organising centre
FDR: False discovery rate
DHC: Dynein heavy chain
ATP1α1: Na^2^^+^/K^+^ ATPase subunit α1
