# Supplementary material for: Affinity-based proteomics reveals novel binding partners for Rab46 in endothelial cells
Source: Sci Rep. 2021 Feb 18;11:4054. doi: 10.1038/s41598-021-83560-y (PMC7893075; doi:10.1038/s41598-021-83560-y)
Supplement: Supplementary file 1 — Supplementary Information 1. [file 41598_2021_83560_MOESM1_ESM.docx]

**Affinity-based proteomics reveals novel binding partners for Rab46 in endothelial cells**

*Lucia Pedicini^1^, Sabina D Wiktor^1^, Katie J Simmons^1^, Ashley Money^1^, Lynn McKeown^1^*^⸹^

**
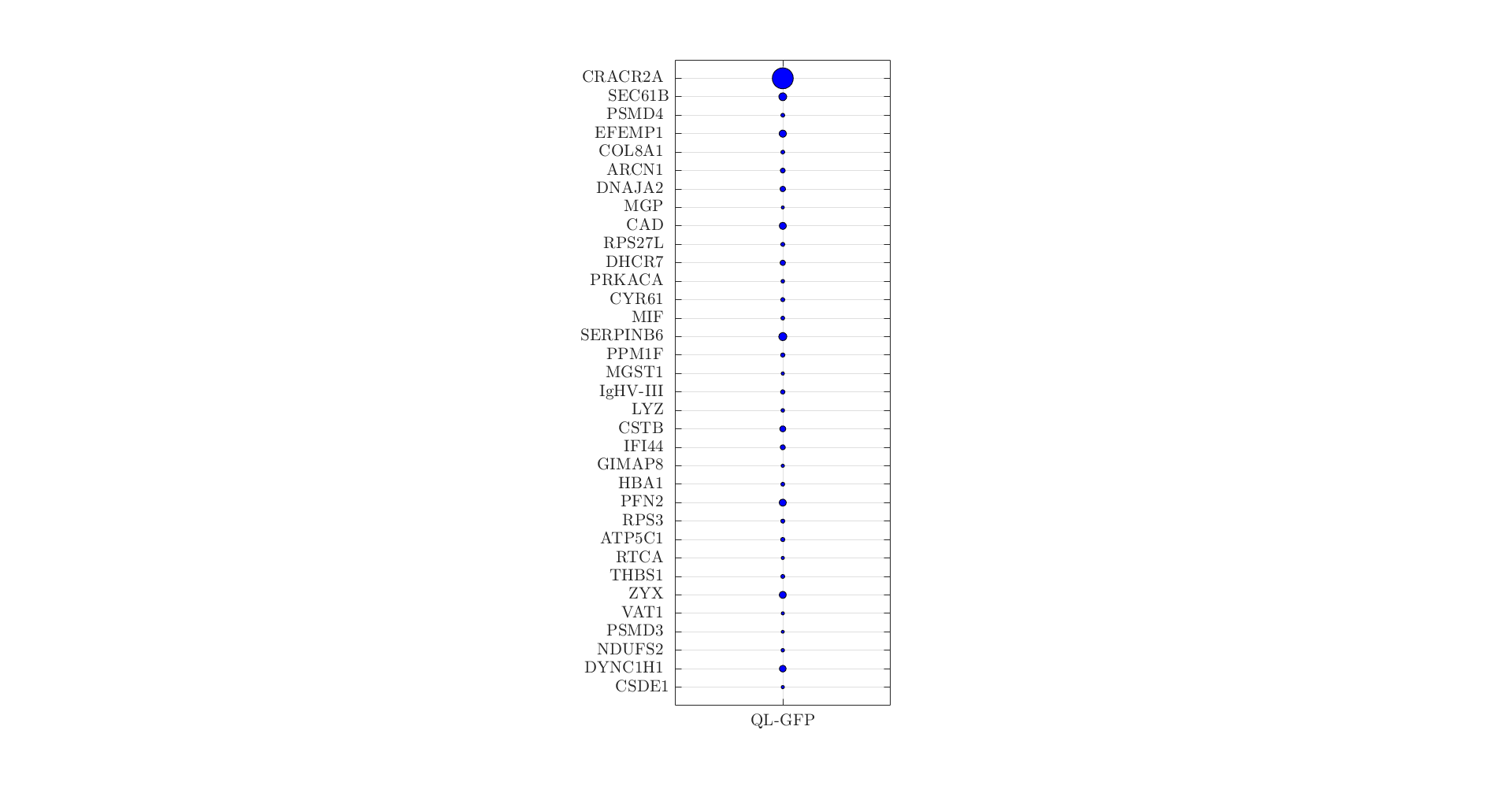
**^1^Leeds Institute of Cardiovascular and Metabolic Medicine, Faculty of Medicine and Health, University of Leeds, Leeds. LS2 9JT.

**Supplementary Figures**

**Fold change**

**Fig. S1: Rab46 enriched proteins compared to GFP control.** Proteins co-precipitate with GFP-Q604L identified by mass spectrometry analysis in reference to GFP control with fold change ≥ 1.5 and significant p-value. Enriched protein are ranked based on changes of fold change and the p-value visualised with a bubble plot where the circle area is proportional to p-values. Bigger circles show smaller (more significant) p-values. Bubble plot created with MATLAB 2018b (https://www.mathworks.com/matlabcentral/fileexchange/48005-bubbleplot-multidimensional-scatter-plots).


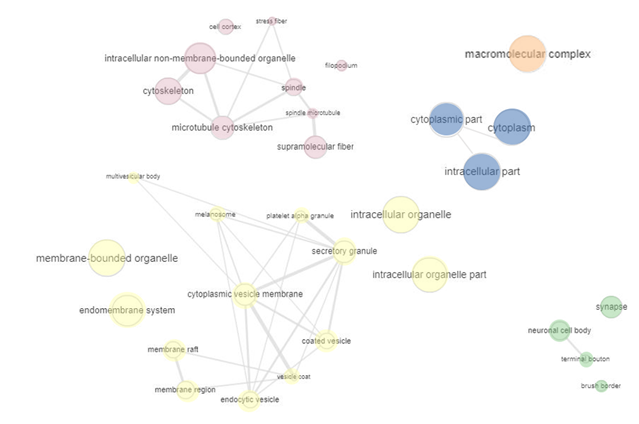

**Fig. S2: GO enrichment analysis elucidating enriched cellular component (CC).** GO terms identified using dataset imported from STRING and analyzed with REVIGO web server. Redundant GO terms were excluded for clarity. Results are visualized with REVIGO interactive maps (http://revigo.irb.hr/) showing enriched enriched CC GO terms. Similar GO terms are colour coded. The colour matches the bar colours of the bar chart on the right showing percentage of protein distribution among the most represented GO terms identified.

**b**


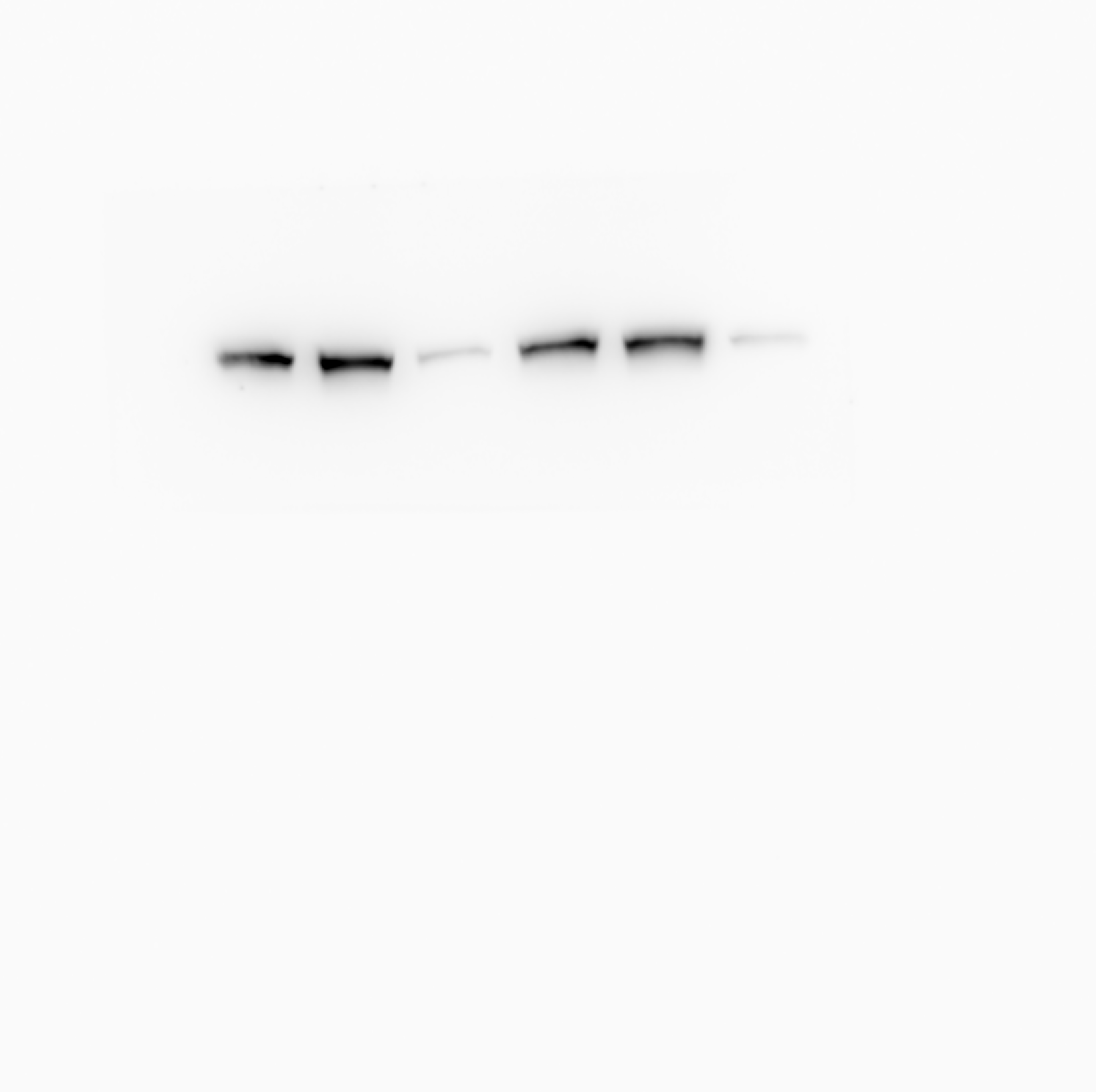

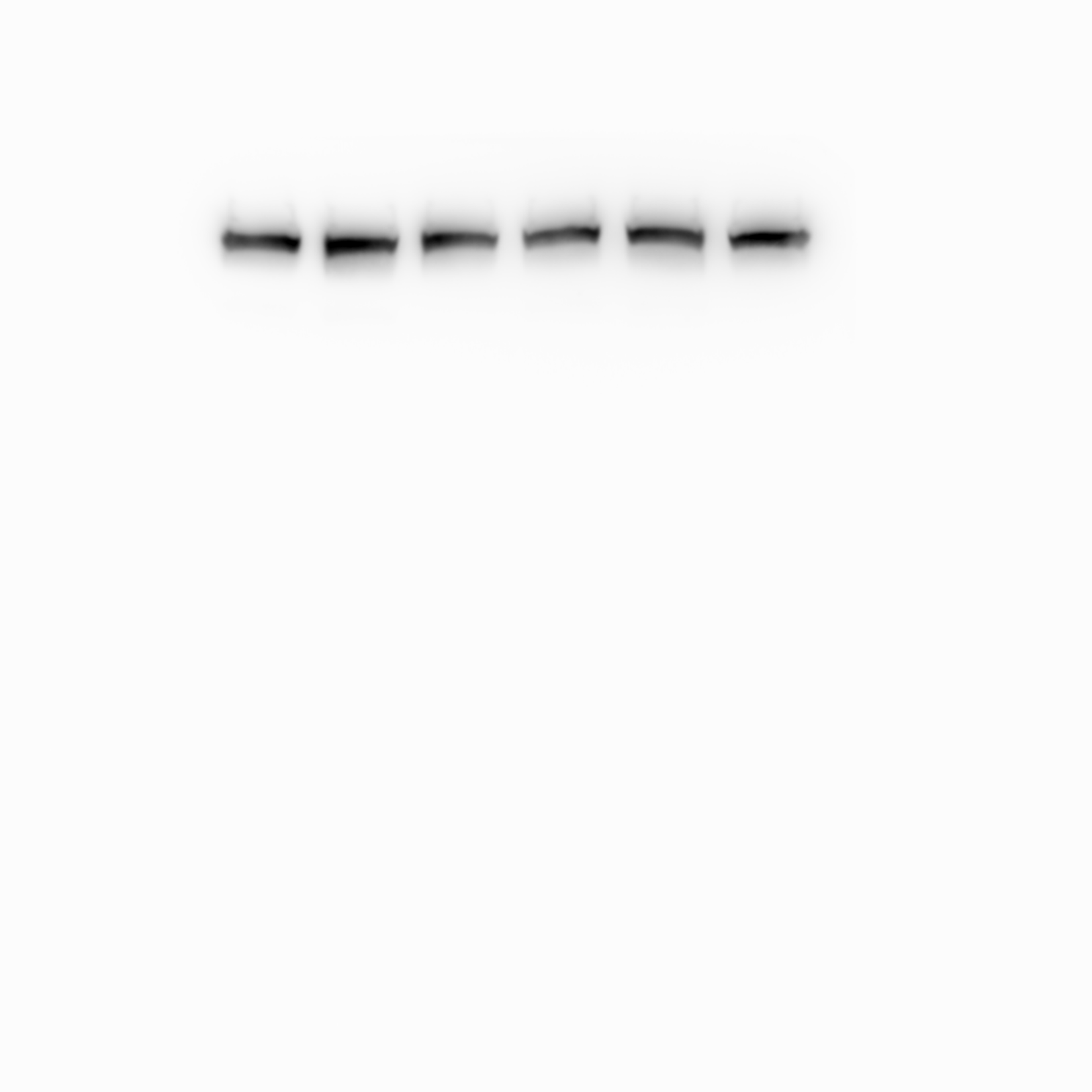


**ATP1α1**

**Vinculin**

**Mock**

**scr. siRNA**

**siRNA**

**110 -**

**120 -**

**kDa**

**a**


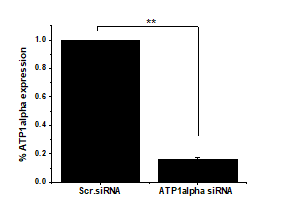


**Fig. S3: Validation of Na^2+^/ K^+^ ATPase subunit α1 antibody.** a) Representative western blot depicting specificity of ATP1α1 antibody. ATP1α1 depleted endothelial cells, transfected with ATP1α1 siRNA (100 nM), show reduced intensity bands compared to control siRNA. Vinculin used as loading control. b) Mean data from 3 biological repeats. Plot created with Origin(Pro), "Version 2019b". OriginLab Corporation, Northampton, MA, USA.


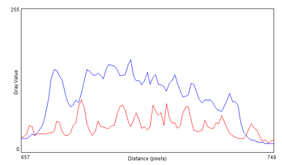

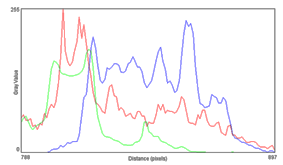


**ATP1α1**

**Q604L**


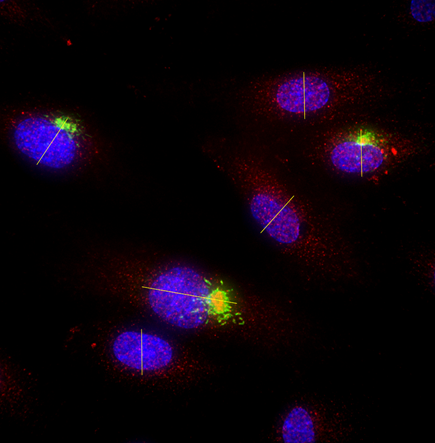


1

2

31

4

3

1


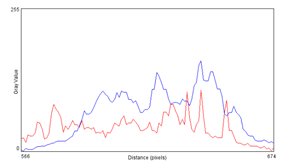

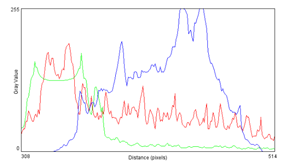


4

2

**Fig S4. Measurements of the cellular distribution of ATP1α1 in Rab46 Q604L transfected cells.** Images depict HUVECs transfected with Rab46 Q604L active mutant (green) and stained for endogenous ATP1α1 (red). Scans from the lines across the nucleus numbered 1-4 are shown in the relevant boxes where the blue lines are DAPI, red ATP1α1 and green Rab46. Fiji ImageJ (Color_Profiler plugin https://imagej.nih.gov/ij/plugins/color-profiler.html) was used to generate RGB line scans. Note the perinuclear localisation of ATP1α1 in cells expressing Q604L compared to the even distribution in cells not expressing Q604L.


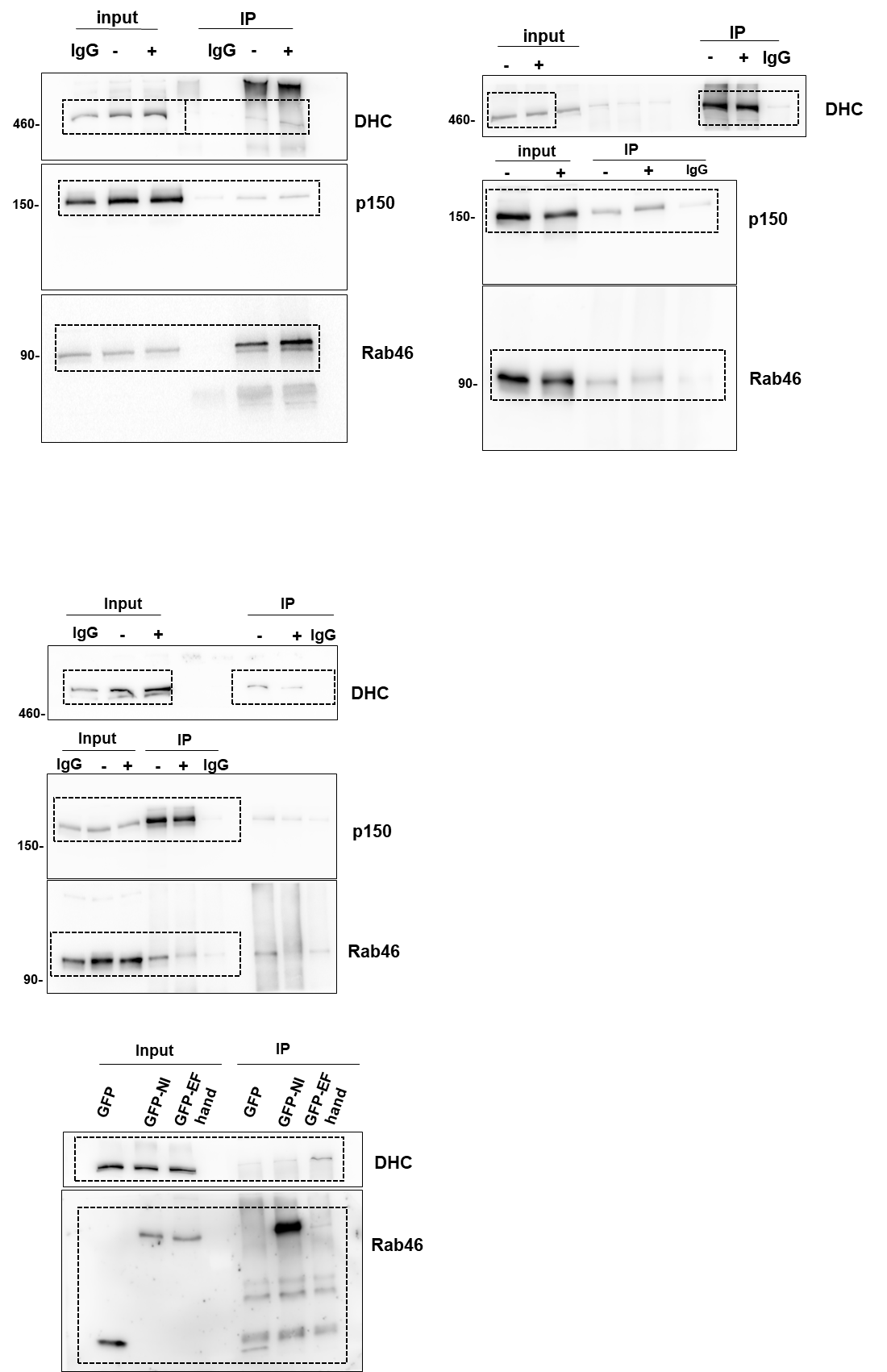


**b**

**d**

**a**


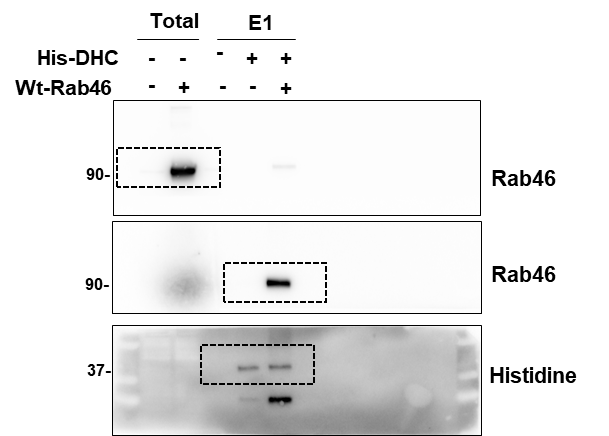


**e**

**c**

**Fig. S5. Full imaged membranes from experiments in figure 3**. a) Fig 3a. b) Fig. 3b. c) Fig. 3c. d) Fig 3d. e) Fig. 3f. Dashed lined rectangles indicate the regions used in the figures.


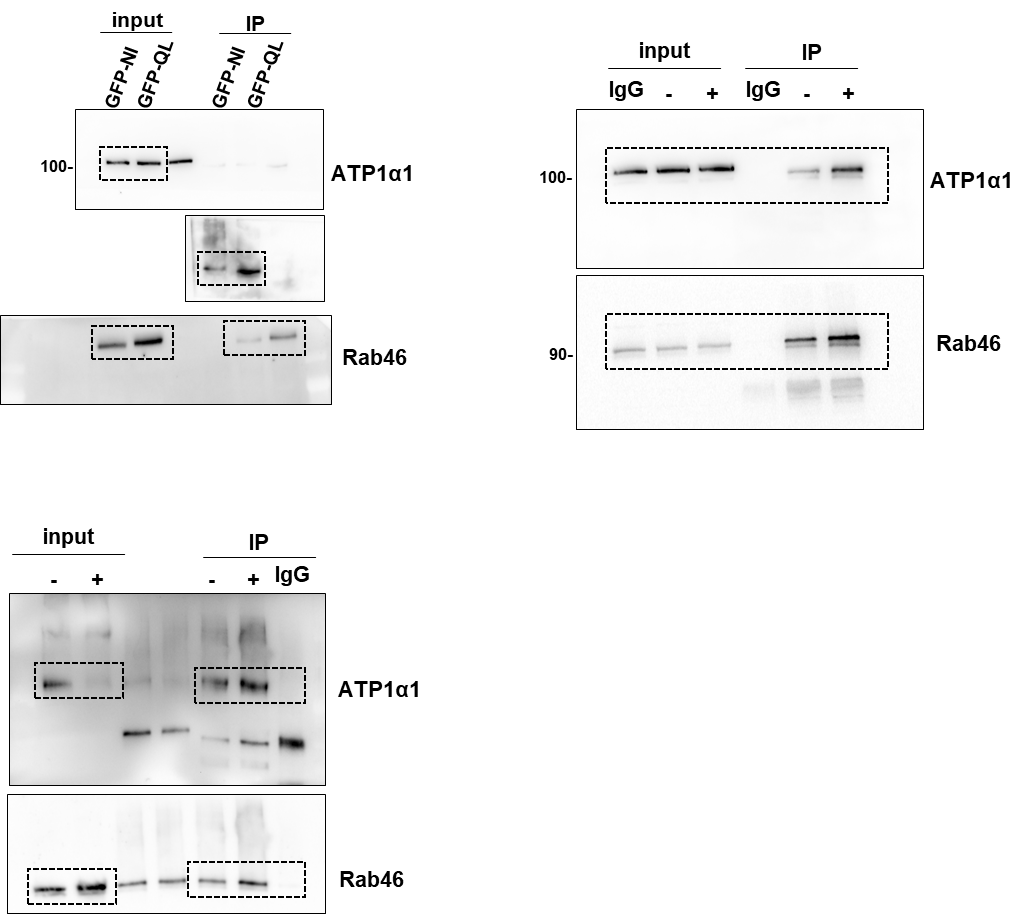


**b**

**c**

**a**

**Fig. S6. Full imaged membranes from experiments in figure 5**. a) Fig 5a. b) Fig. 5b. c) Fig. 5c. Dashed lined rectangles indicate the regions used in the figures.
